# Cryptogenic strokes and neurological symptoms of Fabry disease

**DOI:** 10.3389/fneur.2025.1529267

**Published:** 2025-03-05

**Authors:** Maria Luisa Ruiz-Franco, Beatriz Vélez-Gómez, Patricia Martínez-Sánchez, Remedios Garófano-López, Carlos Gómez-Navarro, Antonio Arjona-Padillo

**Affiliations:** ^1^Stroke Centre, Department of Neurology, Torrecárdenas University Hospital, Almería, Spain; ^2^Faculty of Health Sciences, CEINSA Health Research Centre, University of Almería, Almería, Spain; ^3^Department of Nephrology, Torrecárdenas University Hospital, Almería, Spain; ^4^Family Heart Disease Unit, Department of Cardiology, Torrecárdenas University Hospital, Almería, Spain

**Keywords:** stroke, Fabry disease (FD), Fabry disease – complications, cryptogenic stroke (CS), cryptogenic stroke

## Abstract

**Introduction:**

Fabry disease (FD) is the second most common lysosomal storage disorder. It mainly affects young people. FD can be characterized by neurological symptoms that can occur in both the central and peripheral nervous systems. Cerebrovascular involvement is common in FD and is considered an important cause of cryptogenic strokes. This study aimed to describe the neurological symptoms in patients with FD in general and, specifically, to determine the frequency of association between this disease and cerebrovascular manifestations in our environment.

**Materials and methods:**

This retrospective, observational, cross-sectional study included all patients in the FD registry of the nephrology and cardiology Departments of our center. A descriptive analysis of demographic, neurological, clinical, and neuroimaging variables was performed, with a particular focus on their association with stroke or other cerebrovascular events prior to diagnosis.

**Results:**

A total of 25 patients were included, with 14 (68%) of them being women. The median age of the patients was 52 years (relative intensity of collaboration [RIC] = 24.5). The patients belonged to five families with specific galactosidase alpha gene (GLA) mutations. Neuroimaging was performed in 13 (52%) patients, most of whom did not have neurological symptoms but had normal imaging results. Only 2 (8%) patients had nonspecific white matter hyperintensities. Among the 11 (44%) patients with neurological involvement, the most common symptom was pain in the extremities (32%). Stroke was identified in only one patient (4%), which occurred prior to the diagnosis of FD and was determined to be of cardioembolic etiology.

**Discussion:**

FD is found to be associated with several neurological symptoms. In our study, the most common neurological symptom was limb pain, which had varied characteristics. On the other hand, the incidence of stroke was significantly lower than that expected.

## 1 Introduction

Fabry disease (FD) is the second most common lysosomal storage disorder, following Gaucher disease. This disease is an X-linked inherited disorder of sphingolipid metabolism caused by decreased or absent activity of the lysosomal enzyme *α*-galactosidase. This enzyme typically affects the heart (cardiomyopathy and arrhythmias), kidneys (resulting in proteinuria and renal failure), nervous system (causing neuropathic pain and stroke), and skin (manifesting as angiokeratomas) (1, 2). The prevalence of FD is estimated to be between 1/117000 and 1/476000. However, a comprehensive international database would be necessary to determine the true prevalence (3–6).

Peripheral neuropathy is the most common form of presentation, affecting approximately 80% of patients with FD (7). Cerebrovascular involvement is also frequently observed. FD is considered to be a significant cause of stroke, with ischemic strokes/transient ischemic attacks (TIAs) occurring in up to 25% of patients with FD (7). The pathology of FD increases the risk of stroke across all age groups, particularly in young individuals. It raises the risk of ischemic stroke up to 12 times in men aged 25–44 years and 10 times in women (8). Additionally, FD is also associated with hemorrhagic stroke and other less frequent cerebrovascular manifestations (vascular dementia, cervical artery dissection, etc.).

Magnetic resonance imaging (MRI) is considered the gold standard for imaging. In patients with FD, certain findings that have been described as more frequent (7), such as the pulvinar sign (9), extensive white matter lesions (10), or ectasia and elongation of the basilar artery (11), can be found. However, these findings are not specific to this pathology and must be interpreted in a clinical context. The pulvinar sign is now understood to occur with a significantly lower incidence in Fabry disease than previously described, and selective involvement of the pulvinar is recognized as a rare neuroradiological sign of the disease (12).

This study aims to describe the neurological symptoms in patients with FD in general and, specifically, to assess the frequency of association between this disease and cerebrovascular manifestations in our environment.

## 2 Materials and methods

This retrospective, observational, and cross-sectional study included patients from the Fabry disease registry in the Nephrology and Cardiology Services of Torrecárdenas University Hospital in Almeria. The clinical history (Diraya and Single Health Record) was reviewed to identify neurological symptoms assessed in the emergency department, primary care, or hospital. This review included assessments by the Neurology Service (through consultations or hospital admissions) and complementary tests that were performed (neurophysiology and neuroimaging). To obtain this information, an independent reviewer retrieved the reports containing the necessary data from the patient’s electronic medical records, anonymized these reports, and sent them to the study authors for the collection of variables into the databases. For report selection, the reviewer searched the records of emergency, neurology, nephrology, cardiology, neurophysiology, and imaging studies.

The inclusion criteria for this study were as follows: patients aged 18 years and those with a genetic or biochemical diagnosis of Fabry disease, specifically with pathogenic mutations, in the province of Almería. The exclusion criteria had patients with mutations of uncertain significance or nonpathogenic mutations and those without documented follow-up.

### 2.1 Variables

The study considered several variables, including demographic, clinical, and neuroimaging data. Demographic variables included sex, current age, age at diagnosis of Fabry disease, and disease duration. Clinical variables encompassed ischemic stroke (categorized by large vessel, lacunar, anterior territory, and posterior territory), hemorrhagic stroke (deep territory and lobar), and stroke etiology (atherothrombotic, cardioembolic, arterial dissection, cryptogenic). Other clinical symptoms assessed included vascular dementia, neuropathy (sensory, motor, or mixed), distal extremity pain, acroparesthesia, palmoplantar hypesthesia, dysautonomia (manifested by hypohidrosis, reduced salivation, reduced lacrimation, intestinal motility disorders, and cardiac arrhythmias), as well as other diagnoses such as multiple sclerosis, aseptic meningitis, and dolichoectasia. Cardiac involvement was assessed in terms of cardiomyopathy and arrhythmias, whereas renal involvement was evaluated through proteinuria and renal insufficiency. Cutaneous and ocular involvement was determined by the presence of angiokeratomas and cornea verticillate, respectively. Additional neurological symptoms, such as headache and vertigo, were also recorded. Neuroimaging data included the presence of white matter lesions (classified as periventricular, subcortical, or generalized), the pulvinar sign, and ectasia or elongation of the basilar artery.

### 2.2 Statistical analysis

A descriptive analysis of the variables was performed. Quantitative variables were expressed as means ± standard deviations (SD) or medians (interquartile ranges). Qualitative variables were expressed as total numbers and percentages (%). Statistical analysis was conducted using the Statistical Package for Social Sciences (SPSS), Windows version 27.0 (IBM, Armonk, New York).

## 3 Results

A total of 28 patients were obtained from the records under follow-up by the nephrology and cardiology services, of which three were excluded due to age below 18 years, resulting in a final sample of 25 patients, of whom 14 (68%) were women with a median age of 52 years (RIC = 24.5). The median age at disease diagnosis was 38 years (RIC = 26.5), and the median time of evolution at the time of analysis (May 2024) was 10 years (RIC = 6).

The patients belonged to five families, each with a specific genetic GLA mutation: Family A with the mutation p.Pro205Ser consisted of 16 members (62.5% female), Family B with the mutation p.Val199Gly comprised 2 members (100% female), Family C with the mutation p.Trp626Tre included 4 members (75% female), Family D with the mutation p.Gly80Asp contained 2 members (100% female), and Family E with the mutation p.Cys202Arg consisted of 4 members (75% female).

Of the 25 patients, 13 (52%) had renal involvement, 16 (64%) had cardiac involvement, 6 (24%) had skin involvement, 2 (8%) had ocular involvement, and 11 (44%) had neurological involvement. Neuroimaging studies were performed in 13 patients (52%), most of whom did not have neurological symptoms, and the results were normal in the majority (44%) with non-specific white matter lesions in two patients (one with subcortical predominance and one with periventricular predominance) (Table 1).

**Table 1:** The results for all variables included in the study.

	Total (*n* = 25)
Women, n (%)	17 (68)
Actual Age, median (RIC)	52 (24.5)
Age at diagnosis, median (RIC)	38 (26.5)
Evolution time, median (RIC)	10 (6)
Cardiological involvement, n (%)	16 (64)
Kidney involvement, n (%)	13 (52)
Skin involvement, n (%)	6 (24)
Eye involvement, n (%)	2 (8)
Neurological involvement, n (%)	11 (44)
Neurological assessment	14 (56)
Debut, n (%)	4 (16)
Evolution, n (%)	7 (28)
Stroke, n (%)	1 (4)
Vascular dementia, n (%)	1 (4)
Neuropathy, n (%)	3 (12)
Limb pain, n (%)	8 (32)
Acroparesthesia, n (%)	4 (16)
Dysautonomia, n (%)	4 (16)
Previous neurological diagnosis, n (%)	2 (8)
Other neurological symptoms, n (%)	7 (28)
Neuroimaging, n (%)	13 (52)
Normal, n (%)	11 (44)
Periventricular white matter hyperintensities	1 (4)
Deep white matter hyperintensities	1 (4)
Pulvinar sign	0 (0)
Elongation of the basilar artery	0 (0)

**Figure 1:**
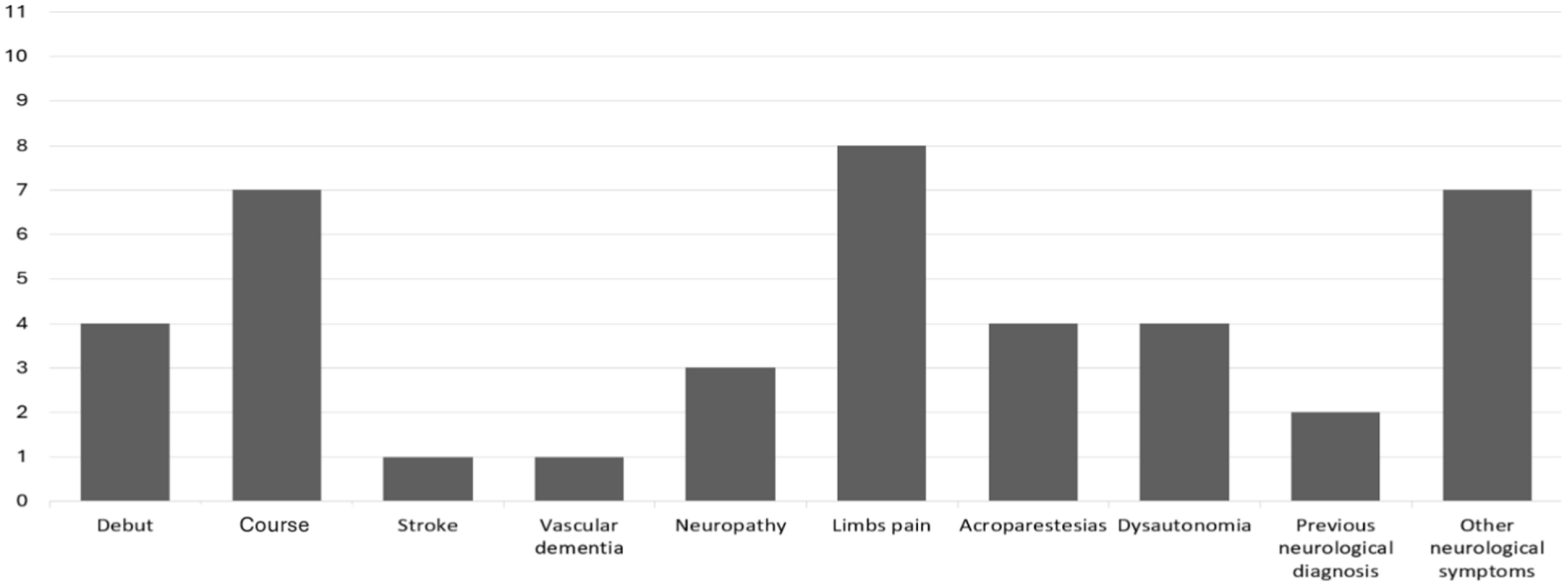
Neurological involvement (n = 11). This graph represents the 11 patients who exhibited neurological involvement, indicating whether it appeared at disease onset (4/11) or during the disease course (7/11), along with the specific type of neurological involvement.

Among the 11 patients with neurological involvement, the majority (63.6%) presented with neurological involvement as the disease progressed. The most frequent symptom was pain in the extremities, with variable characteristics, sometimes in the form of burning and sometimes with radicular distribution, which did not always correlate with pathological findings in neurophysiological studies. The second most frequent symptom was acroparesthesia (36.4%) and dysautonomia, which also appeared in 4 patients (36.4), in the form of hyposweating in 2 of them and in the form of intestinal hypomotility in the other two. Neuropathy confirmed by neurophysiological study was observed in 3 patients (27.3), all of whom had mixed characteristics and were sometimes related to other comorbidities. Vascular dementia was observed in one patient. Another patient had a stroke before diagnosis, which was associated with the presence of *de novo* non-valvular atrial fibrillation. Other neurological symptoms were common among these patients, with headache being the most frequent (20%), typically meeting the migraine criteria. Occipital neuralgia was diagnosed in one patient; headache was diagnosed before the FD diagnosis. Two patients reported vertigo and one with non-specific visual disturbance and dysgeusia (Figure 1).

Of the total sample, 14 (56%) received enzyme replacement therapy (ERT). When the patients were categorized based on the presence or absence of neurological involvement, 5 (45.5%) of the patients with neurological involvement received ERT, whereas the percentage was higher in the group of patients without neurological involvement, 8 (57.1%); however, this difference was not statistically significant.

Furthermore, we analyzed the various neurological and non-neurological clinical manifestations by family and, consequently, by genetic mutation.

First, we defined the system-specific involvement in relation to each genetic mutation, and in the following paragraph, we provided a detailed description of the neurological involvement, also according to the specific genetic mutation. In Family A (mutation GLA p.Pro205Ser), 11 members (68.75%) exhibited cardiac involvement, 9 members (56.25%) presented renal involvement in the form of proteinuria, 3 members (18.75%) had cutaneous involvement, and 5 members (31.25%) displayed neurological involvement. In Family B (mutation GLA p.Val199Gly), 100% of the patients exhibited cutaneous, renal, and ocular involvement, and 1 member (50%) exhibited cardiac and renal involvement. In Family C (mutation nonsense GLA p.Trp626Tre), cardiac and renal involvement was observed in 25% of the patients, 100% had neurological involvement, and none exhibited cutaneous or ocular involvement. In Family D (mutation missense GLA p.Gly80Asp), 1 member (50%) exhibited proteinuria, and no patients presented with involvement in other systems. Finally, in Family E (mutation GLA Cys202Asp), all patients (100%) demonstrated cardiac involvement, 25% had renal and cutaneous involvement, and 50% presented neurological involvement, with no ocular involvement observed in any of them (Table 2).

**Table 2:** Phenotypes and treatment characteristics by families and mutations.

	Family A (Pro205Ser) *n* = 16	Family B (Val199Gly) *n* = 2	Family C (Trp262Tre) *n* = 4	Family D (Gly262Asp) *n* = 2	Family E (Cys202Arg) *n* = 4
System involvement					
Heart	11 (68.75%)	1 (50%)	1 (25%)	0 (0%)	4 (100%)
Renal	9 (56.25%)	1 (50%)	1 (25%)	1 (50%)	1 (25%)
Cutaneous	3 (18.75%)	2 (100%)	0 (0%)	0 (0%)	1 (25%)
Ocular	0 (0%)	2 (100%)	0 (0%)	0 (0%)	0 (0%)
Neurological	5 (31.25%)	2 (100%)	2 (50%)	0 (0%)	1 (25%)
Treatment					
No treatment	9	1 (50%)	0 (0%)	0 (0%)	2 (50%)
Migalastat	3	0 (0%)	0 (0%)	0 (0%)	0 (0%)
Fabrazyme	4	1 (50%)	4 (100%)	0 (0%)	1 (25%)
Replagal	3	0 (0%)	0 (0%)	0 (0%)	1 (25%)
Mean duration of therapy (months)	123	36	56	-	10

In the total sample, 14(%) patients were treated with migalastat, alpha-galactosidase, beta-galactosidase, or a combination of these medications (Table 2). When divided by family, of the 16 members of Family A, one was treated with migalastat for 26 months without changes, one received alpha-galactosidase for 138 months without changes, and two were treated with beta-galactosidase for 86 and 160 months, respectively, without changes. The remaining four patients from this family underwent treatment modifications over time: one started with alpha-galactosidase for 120 months and later switched to beta-galactosidase for 26 months; another started with alpha-galactosidase for 108 months, followed by beta-galactosidase for 84 months, and finally migalastat for 24 months. The last member of this family had been administered with beta-galactosidase for 48 months and later switched to migalastat, which was administered for 42 months. In Family B, one of the two patients received treatment with beta-galactosidase for 36 months. In Family C, four patients received beta-galactosidase treatment for 103, 24, 48, and 48 months, respectively. Finally, in Family E, one patient received beta-galactosidase for 14 months, and another received alpha-galactosidase for 6 months. No patient in Family D received treatment.

**Figure 2:**
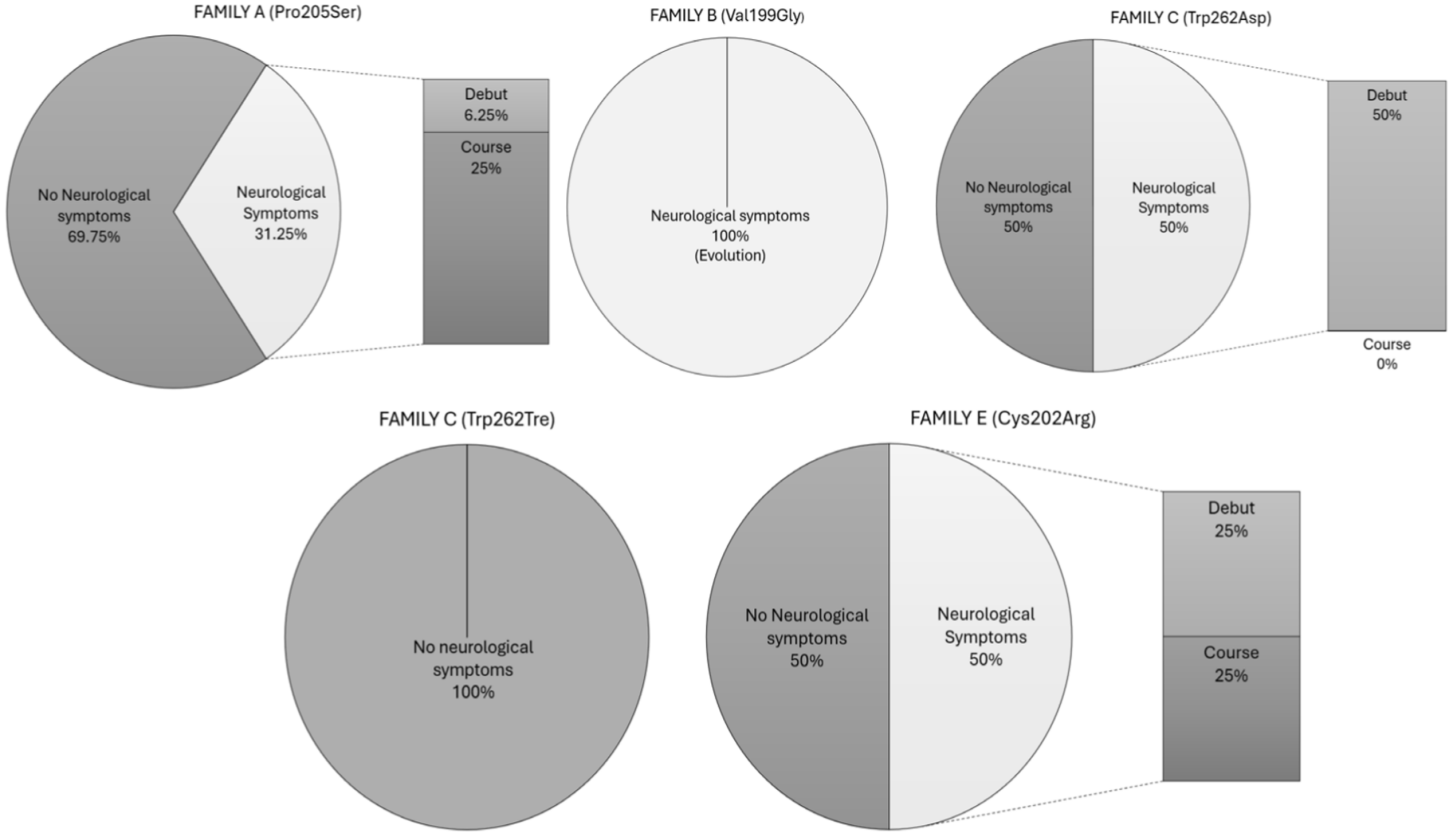
Neurological symptoms by families. This graph shows the percentage of neurological and non-neurological involvement per genetic mutation, indicating the proportion of neurological symptoms that appeared at disease onset or during the course of the disease.

Finally, we analyzed the neurological involvement of the members of each family (Figure 2).

- Family A: Among the five members with neurological symptoms, four exhibited these symptoms during the course of the disease. One patient presented with sensory neuropathy characterized by pain in the extremities, two experienced headaches, and the last had vertiginous symptoms. The patient with sensory neuropathy showed periventricular white matter hyperintensities on MRI and developed vascular dementia, whereas the others had no significant findings on neuroimaging. The fifth patient in Family A exhibited neurological symptoms at disease onset, presenting with pain in the extremities and acroparesthesia. The patient had been evaluated by a neurologist several years previously and was discharged with a diagnosis of headache. MRI revealed the following bifrontal deep white matter hyperintensities:

- Family B: Both members (100%) experienced neurological symptoms throughout the course of the disease, both in the form of neuropathy: one sensory and the other mixed, with the latter being more symptomatic, presenting not only paresthesias but also hypo-anhidrosis and abdominal pain crises. Neuroimaging in both patients was normal.

- Family C: Two of the four patients presented with neurological symptoms. They consisted of one man and one woman, both with extremity pain. No imaging studies were available for either of them.

- Family D: No patient exhibited neurological symptoms.

- Family E: Of the total, 50% (2 out of 4) presented with neurological symptoms. One patient experienced headaches and vertigo throughout the course of the disease and had a prior diagnosis of occipital neuralgia before Fabry’s disease was detected. The second patient in the sample was the only one to have a stroke. He had experienced a transient ischemic attack (TIA) 4 years previously and ultimately suffered a large vessel ischemic stroke in the posterior circulation, also in the context of non-valvular atrial fibrillation. During the etiological study of this stroke, ventricular hypertrophy was found on echocardiography, prompting further investigation. He was diagnosed with Fabry disease 1 year later (Table 2).

## 4 Discussion

FD is associated with several neurological symptoms. In the current study, the most frequent neurological symptom was limb pain with varied characteristics, with a lower prevalence of cryptogenic stroke than expected. These results suggest that the prevalence of stroke among patients with Fabry disease may be overestimated, in contrast to the majority of previous studies reporting a high prevalence of this complication.

The most frequent neurological symptom among our sample was limb pain. This is congruent with the available literature: peripheral neuropathy is the most common presentation (80%) in the form of fine fiber sensory neuropathy in which distal pain is the most frequent presentation (60–80%), followed by acroparesthesia and hypoaesthesia in the hands and soles of the feet. When it appears in childhood, this symptomatology should prompt us to consider this entity (7).

On the other hand, the low frequency of debuts with strokes was higher than expected. According to available evidence, the frequency of vascular events in patients with FD is >25% (7). Studies analyzing this relationship vary depending on the population: some analyze “cryptogenic stroke patients” and look for FD, and others analyze “patients with FD” and look for stroke as a manifestation.

The most frequent analyses are the cryptogenic stroke populations and the frequency of FD among them. We identified 14 studies with these characteristics (13–24). The majority of these studies are retrospective, and the frequency of FD varies between 0 and 6.49%. Some studies found a notable presence of FD, such as Rolfs et al. (13) with 3.88% of cases, while others reported very low prevalence, such as Kinoshita et al. (23) and Reinsin et al. (24), who found rates of 0.3 and 0.16%, respectively. The highest observed prevalence was reported by Wolkiak et al. (6.49%) (15) and Gündoğdu et al. (3.7%) (19). Romani et al. (25) recently analyzed the largest sample size (1906 patients) in a multicenter study involving 33 Italian neurological stroke units and found a prevalence of FD of 3 (0.16%).

In our center, we analyzed the frequency of FD in 99 young individuals with stroke, without identifying any positive cases during a 19-month observation period (26). As a result, our diagnostic protocol currently includes FD screening only in the presence of other suspicious manifestations (non-hypertensive hypertrophic cardiomyopathy, proteinuria, renal failure, neuropathic pain, angiokeratomas, megadolichobasilar, pulvinar sign, white matter hyperintensities of undetermined etiology, angiokeratomas, cornea verticillata) and not as a standard procedure.

Two systematic reviews analyzed studies about FD among cryptogenic stroke patients. The first study from 2013 included 9 studies and found that FD may explain 1% of strokes among young people and between 3 and 5% of cryptogenic strokes (27). The second systematic review, which was more recent from 2021, included 11 studies (28). In their pooled analysis, the prevalence of FD was 0–3.88%, and the results suggested that this prevalence is higher in patients with stroke recurrence.

On the other hand, we found 4 studies about the neurological manifestations of FD, similar to our study (29–32). First, Buechner et al. (29) studied the central nervous system involvement in a group of FRD Italian patients and described stroke in 25.6%. In the second study, Sims et al. (30) analyzed the Fabry Registry data to identify patients who suffered during the natural history period and found a prevalence of 5.6%. Afterward, Schelleckes et al. (31) detected 5 vascular events (2 strokes and 3 transient ischemic attacks) among 15 patients with FD. More recently, a multicenter study with a larger sample size (54 patients) described 5 strokes (9.25%) among them (32). This is consistent with our study, in which the prevalence (4%) was even lower than the lowest figure reported in the cited articles. Since ERT has been shown to ameliorate endothelial dysfunction in Fabry patients (7), we hypothesize that the difference in stroke frequency between studies may be influenced by whether or not patients initiated treatment: in the study with the highest prevalence of stroke detected (33), no patient had received enzyme replacement therapy (ERT), while 14 (56%) of our sample were undergoing ERT. However, in the studies by Sims and Nampoothiri, none of the patients received ERT (34).

Regarding genetics, understanding the phenotypic correlations of all known GLA gene variants and elucidating the pathophysiological mechanisms that link genetic mutations to their clinical manifestations are crucial for all stakeholders involved in providing healthcare to patients with Fabry disease and their families (34). The Human Gene Mutation Database (35) reports more than 900 GLA gene variants, of which nearly 75% are point mutations, most of which are pathogenic. The disease exhibits an X-linked recessive inheritance pattern. It is associated with mutations in the GLA gene (locus Xq22.11) in nearly 100% of the affected males, accompanied by a reduction in the enzymatic activity of Alpha-Galactosidase A. The clinical presentation encompasses a broad spectrum, ranging from mild in heterozygous females to severe in hemizygous males affected by the classic form, characterized by absent residual alpha-galactosidase activity.

Our study included five missense/nonsense mutations, one per family: GLA p.P205S, GLA p.V199G, GLA p.W626X, GLA p.G80D, and GLA C202A; we found a bibliography about all of them (36–40).

In Family A (GLA p.P205S), two patients exhibited periventricular white matter hyperintensities on MRI. A Chinese study aimed to evaluate the genotype–phenotype correlation in patients with Fabry disease, including the nonsense mutation Pro205Ser (37). This mutation was associated with an atypical presentation predominantly affecting the kidneys without white matter hyperintensities.

The only patient in the sample who presented with a stroke belonged to Family E. This mutation (Cys202Arg) is listed in ClinVar (633244), HGMD (CM1826087), and dbSNP (rs1569303843) as a pathogenic mutation causing Fabry disease. It was first described in the literature in a case report of cardiac involvement (41). These findings are consistent with the clinical presentation of our patient with cardioembolic stroke who exhibited this mutation and ventricular hypertrophy.

In neuroimaging studies, although characteristic findings are observed in Fabry disease (FD) patients, most of these are nonspecific, such as the pulvinar sign (12). The identification of corpus callosum lesions is particularly valuable for differentiating between multiple sclerosis (MS) and FD, given that patients with FD demonstrate a significantly lower incidence of corpus callosum involvement. This approach may help clinicians to promptly establish an accurate diagnosis and develop appropriate management strategies (42). The limitations of our study stem from its retrospective nature and low prevalence of vascular manifestation.

The neurological manifestations of FD are variable. The most common symptom in our population was limb pain, according to the literature. On the other hand, in our environment, the low frequency of ischemic stroke before and after diagnosis is important compared to the expected frequency. Nevertheless, the prevalence varies among studies because of the selected population and may be overestimated in some of them. FD is a possibly treatable because of cryptogenic stroke that should be considered, particularly in young patients with cardiopathy or proteinuria. The actual prevalence of FD among cryptogenic stroke patients can vary geographically according to genetic mutations and should be analyzed in future studies to determine if the prevalence is overestimated.

In conclusion, FD is associated with several neurological symptoms. In our study, the most frequent neurological symptom was limb pain with varied characteristics. On the other hand, the low frequency of stroke was important compared to that expected.

## Data Availability

The original contributions presented in the study are included in the article/supplementary material, further inquiries can be directed to the corresponding author.
